# Agreement of Clinician‐Administered and Modified Parent‐Administered House‐Brackmann Scales in Children with Bell's Palsy

**DOI:** 10.1002/oto2.44

**Published:** 2023-03-24

**Authors:** Franz E. Babl, Nitaa Eapen, David Herd, Meredith L. Borland, Amit Kochar, Michael Zhang, Ed Oakley, Sandy M. Hopper, Robert G. Berkowitz, Catherine L. Wilson, Amanda Williams, Mark T. Mackay, Katherine J. Lee, Stephen Hearps, the PREDICT (Paediatric Research in Emergency Departments International Collaborative) research network

**Affiliations:** ^1^ Emergency Department Royal Children's Hospital Parkville Victoria Australia; ^2^ Clinical Sciences, Murdoch Children's Research Institute Parkville Victoria Australia; ^3^ Department of Paediatrics, Faculty of Medicine, Dentistry and Health Sciences University of Melbourne Parkville Victoria Australia; ^4^ Department of Critical Care, Faculty of Medicine, Dentistry and Health Sciences University of Melbourne Parkville Victoria Australia; ^5^ Emergency Department Queensland Children's Hospital Brisbane Queensland Australia; ^6^ Child Health Research Centre University of Queensland Brisbane Queensland Australia; ^7^ Mater Research Institute Brisbane Queensland Australia; ^8^ Emergency Department Perth Children's Hospital Perth Australia; ^9^ Divisions of Emergency Medicine and Paediatrics University of Western Australia Perth Western Australia Australia; ^10^ Emergency Department Women's and Children's Hospital Adelaide South Australia Australia; ^11^ Emergency Department John Hunter Hospital Newcastle New South Wales Australia; ^12^ Department of Otolaryngology Royal Children's Hospital Parkville Victoria Australia; ^13^ Department of Neurology Royal Children's Hospital Parkville Victoria Australia; ^14^ Melbourne Children's Trial Centre, Clinical Epidemiology and Biostatistics Unit Murdoch Children's Research Institute Victoria Parkville Australia

**Keywords:** Bell's palsy, emergency department, House‐Brackman scale, multicenter trial

## Abstract

**Objective:**

Currently there is no parent administered scale for facial nerve function in children. We set out to assess the agreement between a newly developed parent‐administered modified version of the House‐Brackmann (HB) scale and the standard clinician‐administered HB scale in children with Bell's palsy.

**Study Design:**

Secondary analysis of a triple‐blind, randomized, placebo‐controlled trial of corticosteroids to treat idiopathic facial paralysis (Bell's palsy) in children (6 months to <18 years).

**Setting:**

Multicenter study at pediatric hospitals with recruitment in emergency departments.

**Methods:**

Children were recruited within 72 hours of symptom onset and assessed using the clinician‐administered and the parent‐administered modified HB scales at baseline, and at 1, 3, and 6 months until recovered. Agreement between the 2 scales was assessed using intraclass coefficient (ICC) and a Bland‐Altman plot.

**Results:**

Data were available for 174 of the 187 children randomized from at least 1 study time point. The mean ICC between clinician and parent HB scores across all time points was 0.88 (95% confidence interval, CI: 0.86, 0.90). The ICC for the data collected at baseline was 0.53 (95% CI: 0.43, 0.64), at 1 month was 0.88 (95% CI: 0.84, 0.91), at 3 months was 0.80 (95% CI: 0.71, 0.87) and at 6 months was 0.73 (95% CI: 0.47, 0.89). A Bland‐Altman plot indicated a mean difference between the 2 scores (clinician‐reported minus parent‐reported) of only −0.07 (95% limits of agreement −1.37 to 1.23).

**Conclusion:**

There was good agreement between the modified parent‐administered and the clinician‐administered HB scales.

Acute facial nerve palsy (FNP) is a relatively common condition in children,^1^, ^2^ with most cases idiopathic and referred to as Bell's palsy.^3^, ^4^ FNP has functional, cosmetic, and psychosocial implications for affected children.^5^, ^6^, ^7^ The degree of facial impairment is monitored by clinicians to identify those at risk of prolonged or permanent facial weakness.^8^

The most commonly used clinician administered clinical assessment tool used in children is the clinician‐administered House‐Brackmann scale, which grossly divides patients into 6 grades based on the severity of facial paralysis (Figure 1).^9^, ^10^ However, regular follow‐up assessments with a clinician may not be feasible for a number of reasons such as cost, distance, clinician availability, and other reasons (eg, Covid‐19‐related travel limitations).

**Figure 1:**
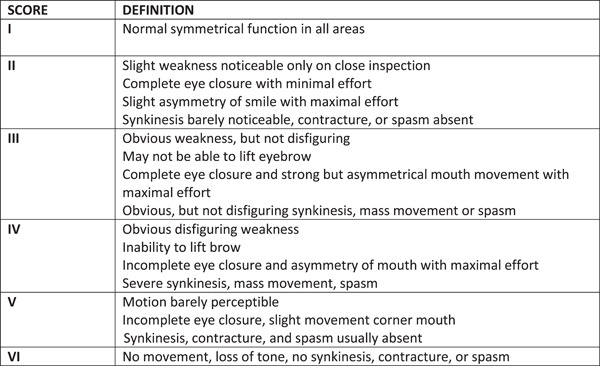
House‐Brackmann grading system adapted by Sullivan et al.^9^, ^10^ Clinician‐administered House‐Brackmann scale.

Parent assessment is already being used either in combination with or as an alternative to clinician assessment in some pediatric conditions.^11^, ^12^, ^13^ As part of a body of work to improve care for children with FNP, we have developed a modified version of the House‐Brackmann scale for parents (parent House‐Brackmann scale), which removed or exchanged medical terms that were not intuitively understandable (Figure 2).^2^, ^4^, ^14^, ^15^, ^16^, ^17^ We recently completed a placebo‐controlled randomized trial in children with Bell's palsy, indicating that prednisolone is not associated with improved recovery of complete facial function at 1 month.^4^ The primary outcome in this study, complete recovery at 1 month, was measured using the clinician‐administered House‐Brackmann scale, as was severity of the FNP at baseline and at 1, 3, and 6 months. At each time point, parents were given the modified parent House‐Brackmann scale to administer themselves. The aim of the current study was to investigate the agreement between the clinician‐administered House‐Brackmann scale and the newly developed and concurrently performed parent‐administered House Brackmann scale. We hypothesize that a modified, parent administered House‐Brackmann scale is comparable to a clinician administered scale. We also explored if the age of the child was associated with the level of agreement between the parent and clinician reports.

**Figure 2:**
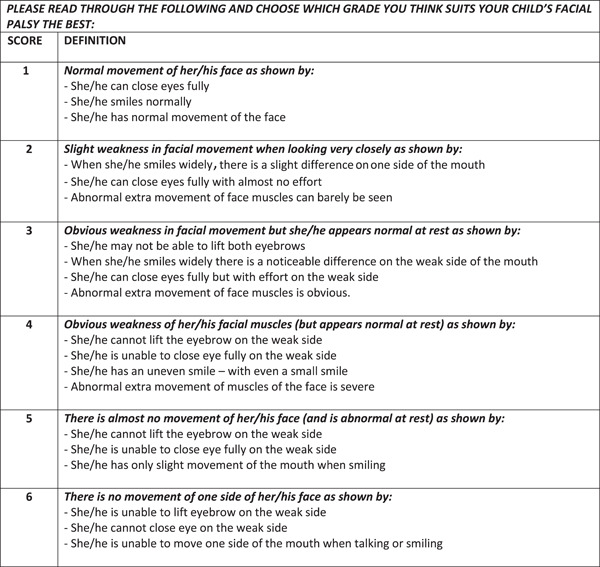
Modified parent‐administered House‐Brackmann grading system. Newly developed modified descriptions for parent‐administered House‐Brackmann scale.

## Methods

### Study Design

Data on facial function scales from clinicians and parents for the current study come from our phase III, triple‐blinded, randomized, placebo‐controlled trial of prednisolone for the treatment of Bell's palsy in children.^4^, ^16^ This study was conducted in 11 emergency departments (EDs) in the Paediatric Research in Emergency Departments International Collaborative (PREDICT) research network in Australia and New Zealand between October 13, 2015, and August 23, 2020. The study protocol has been published with relevant details extracted below.^16^ The trial was approved by the lead institutional ethics committee at the Royal Children's Hospital, Melbourne, Australia (HREC/15/RCHM/V4), and received institutional approval by the research office at each participating site. The study was registered with the Australian New Zealand Clinical Trials Registry (ACTRN12615000563561), registered June 1, 2015, https://www.anzctr.org.au/Trial/Registration/TrialReview.aspx?id=368505&isReview=true.

### Study Population

In brief, children were eligible if aged between 6 months to <18 years and presented within 72 hours of onset of clinician diagnosed Bell's palsy to a participating ED. The exclusion criteria included children with contraindications to prednisolone including active or latent tuberculosis or fungal infection, hypersensitivity to prednisolone, diabetes mellitus, peptic ulcer, and current or past oncological diagnosis. Children were also excluded if there was known otitis media or facial trauma within 1 week prior to onset of symptoms or a prior episode of Bell's palsy.

### Facial Function Scales Used

Clinicians used the House‐Brackmann scale to assess facial function. This scale grossly divides patients with facial paralysis into 6 grades (I‐VI): I represents normal facial function, VI is total paralysis and grades in between correspond to progressively more severe descriptions of facial asymmetry and facial muscle dysfunction, at rest and during movement. For assessments in this study, we used a House‐Brackmann scale version by Sullivan et al,^9^, ^10^ which contains slight variations in word order and presentation to improve the organization and ease of administration of the scale (Figure 1).

We also created a modified version of the House‐Brackmann scale which replaced certain medical terms with lay language to be used by parents (Figure 2), for example, we replaced the term “synkinesis” with “abnormal extra movement of the muscles of the face” prior to commencing the randomized controlled trial.^16^

### Study Procedures

All children suspected of Bell's palsy were assessed for eligibility by their treating clinician. Following informed consent, demographic and other relevant clinical data relating to presentation and past medical history were collected. Treating clinicians in the ED and parents evaluated the severity of facial palsy using the standard House‐Brackmann scale and the modified parent‐administered House‐Brackmann scale, respectively. One month after randomization, participants were reviewed by a specialist clinician (a pediatric neurologist, otolaryngologist, pediatrician, or emergency physician according to site specific resources) and the facial palsy was again assessed using the standard House‐Brackmann scale. Similarly, parents again assessed facial deficit using the parent‐administered House‐Brackmann scale. A further follow‐up assessment at 3 and 6 months was arranged for participants deemed at their previous visit not to be completely recovered (defined in the primary study as a House‐Brackmann score greater than 1) with assessment of facial function as set out above.

In this secondary analysis, the agreement between clinician‐ and parent‐administered House‐Brackmann scales was analyzed collectively across all time points, and separately by time point using the data collected at baseline and the 3 follow‐up visits. Only children assessed using both scales at a single time point were included in the analysis, with children missing an assessment on either scale excluded from the analysis for the affected time point.

### Statistical Analysis

The majority of the analysis for this paper combines participants across the 2 arms. Descriptive statistics were used to report on demographic and baseline characteristics of children enrolled, with median and interquartile range (IQR) reported for continuous measures (due to non‐normality), and frequencies and percentages for categorical variables. The relationships between scores on the clinician‐administered House‐Brackmann and parent‐administered House‐Brackmann scales were illustrated using frequency‐weighted scatterplots. We assessed agreement between the clinician and parent rated scales using the intraclass coefficient (ICC) estimated using linear mixed models (LMM). Four LMM were used to estimate the ICC at each timepoint (baseline, and 1, 3 and 6 months), and a single LMM applied to all data, including an additional random effect for each timepoint, was used to estimate an overall ICC. Agreement was also assessed using a Bland‐Altman plot combining the data from all time points (where both House‐Brackmann scores were rescaled to a score between 0 and 1). All analyses were repeated for children aged <12 years and those aged ≥12 years, as set out in the prespecified Statistical Analysis Plan.^17^

We also repeated the whole group in a post‐hoc sensitivity analysis where we excluded those with a clinician‐rated House‐Brackmann score of 1. This was because at the later time points, it was expected that a large proportion would be completely recovered (House‐Brackmann score = 1), which could obscure a lack of agreement.

## Results

Data from 174 of the 187 participants randomized in the original study were available for this secondary analysis. Thirteen children were excluded because they were missing either parent or clinician‐reported assessment at all follow‐up time points. The baseline characteristics of the study participants are presented in Table 1. In the 174 included children with Bell's palsy, the median age was 10.3 (IQR: 5.1, 13.1) years, and 51% (88 of 174) were female. Most children presented within 48 hours to ED (148 of 174, 86%), with a median clinician‐administered House‐Brackmann grade of 3.5 (IQR: 3‐4) and a parent‐administered House‐Brackmann grade of 4 (IQR: 3‐4) at baseline.

**Table 1:** Baseline characteristics of children with at least one concurrent measure of clinician‐administered and parent‐administered House‐Brackmann scale combining participants in the prednisolone and placebo groups.

	Total cohort		
Child age, y, N Mdn (IQR)	174	10.3	(5.1, 13.1)
Child age, N n (%)			
6 mo to <12 y	174	117	(67.2)
12‐<18 y	174	57	(32.8)
Child sex, N n (%)			
Male	174	86	(49.4)
Female	174	88	(50.6)
Ethnicity, N n (%)			
Nonindigenous	174	164	(94.3)
Indigenous	174	10	(5.8)
Median child weight, kg, N Mdn (IQR)	173	40.0	(20.5, 56.3)
Median length of illness, h, N Mdn (IQR)	168	24.0	(11.0, 48.0)
Length of illness, h, N n (%)			
0‐24	173	77	(44.5)
>24‐48	173	71	(41.0)
>48‐72	173	25	(14.5)
Side of facial weakness, N n (%)			
Left	174	93	(53.4)
Right	174	81	(46.6)
Symptoms on presentation			
House‐Brackmann, N n (%)	174	154	(88.5)
Nonsevere (II‐IV)	174	20	(11.5)
Severe (V and VI)			
House‐Brackmann score—Clinician, N Mdn IQR	174	3.5	(3.0, 4.0)
House‐Brackmann score—Parent, N Mdn IQR	172	4.0	(3.0, 4.0)

Abbreviations: IQR, interquartile range; Mdn, median; N, number with available data; n, number with the given characteristic.

**Figure 3:**
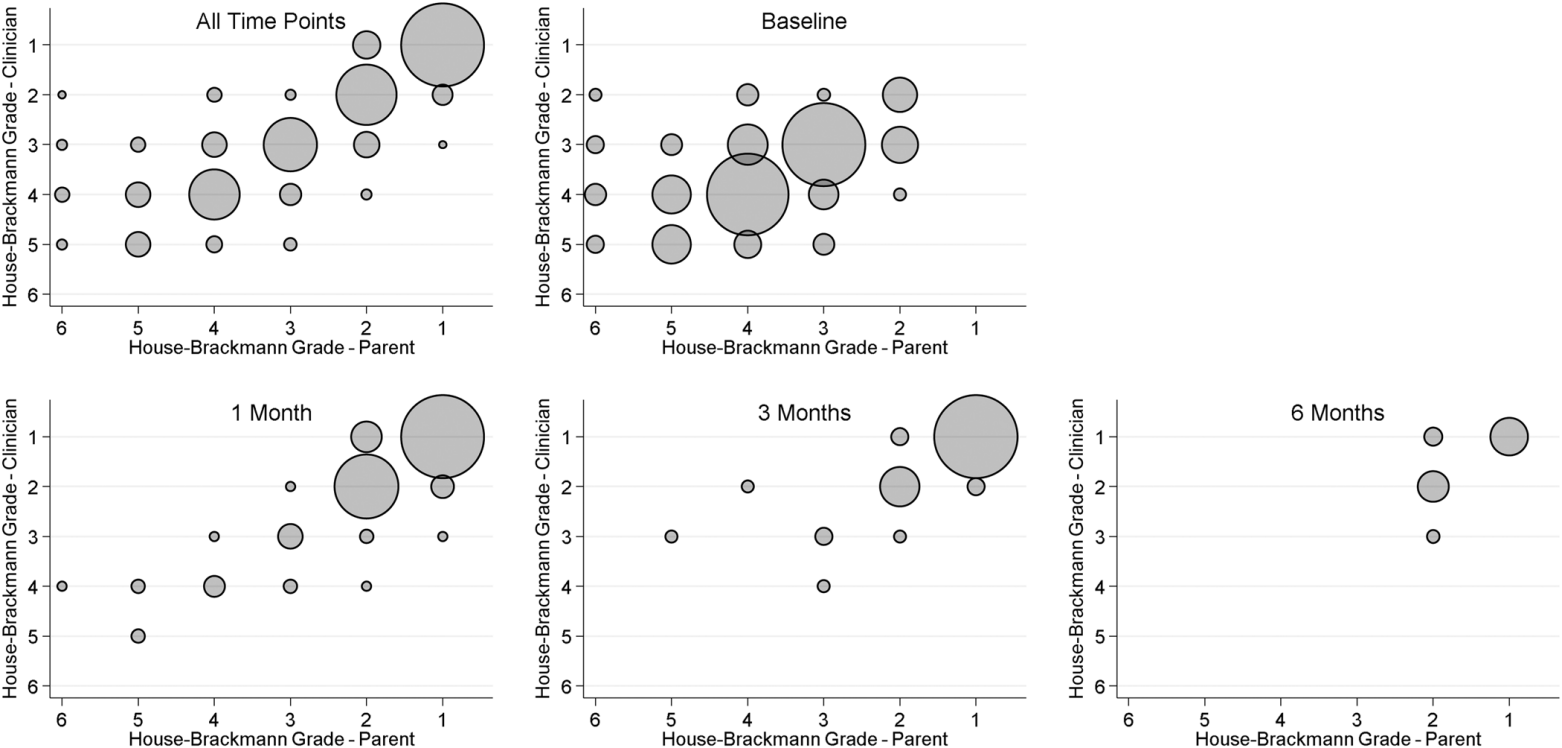
Scatterplot of clinician‐ versus parent‐administered House‐Brackmann scores. The size of the circles are proportional to the frequency that the given combination of scores was observed.

Figure 3 shows a scatterplot of the clinician‐administered House‐Brackmann scores and parent‐administered House‐Brackmann scores overall and at individual timepoints, with circle sizes representing frequency weights. There appears to be greater variability in the clinician‐administered and parent‐administered House‐Brackmann scores at baseline compared to 1 and 3 months. Interpretation of the variability in clinician‐administered and parent‐administered scores is difficult to assess at 6 months due to the small number of observations.

Overall, there was good agreement between the clinician‐administered and parent‐administered House‐Brackmann scores (ICC: 0.88 (95% CI, 0.86, 0.90)) (Table 2). At baseline, there was poor agreement between the scales (ICC 0.53 (95% 0.43, 0.64)), however, this improved at 1 month (ICC: 0.88 (95% CI 0.84, 0.91)), 3 months (ICC: 0.80 (95% CI: 0.71, 0.87)), and 6 months (ICC: 0.73 (95% CI: 0.47, 0.89)). The ICC between the 2 scales in children aged <12 years using data from all of the time points was 0.87 (95%: 0.83, 0.91), and 0.89 (95% CI: 0.85, 0.932) in those aged ≥12 years. The subgroup analysis by age showed a similar pattern of poor agreement at baseline assessment improving at subsequent follow up visits in both age groups.

**Table 2:** Agreement between clinician‐administered and parent‐administered House‐Brackmann scores overall and at each follow up visit

	Total sample			Aged < 12 y			Aged ≥ 12 y		
	N	ICC	(95% CI)	N	ICC	(95% CI)	N	ICC	(95% CI)
All time points	174	0.88	(0.86, 0.90)	117	0.87	(0.83, 0.91)	57	0.89	(0.85, 0.92)
Baseline	172	0.53	(0.43, 0.64)	117	0.50	(0.36, 0.63)	55	0.60	(0.42, 0.75)
1 mo	172	0.88	(0.84, 0.91)	117	0.87	(0.81, 0.90)	55	0.91	(0.85, 0.95)
3 mo	70	0.80	(0.71, 0.87)	46	0.84	(0.73, 0.91)	24	0.77	(0.58, 0.89)
6 mo	18	0.73	(0.47, 0.89)	11	0.72	(0.38, 0.91)	7	0.69	(0.26, 0.93)

Abbreviations: CI, confidence interval; ICC, intraclass correlation coefficient.

In the post‐hoc analysis, removing children who scored House‐Brackmann I on the clinician scale (Table 3), the ICC reduced to 0.73 (95% CI: 0.67, 0.78). A similar ICC was observed in children aged <12 years (ICC 0.72 (95% CI: 0.65, 0.78)) and children aged ≥12 years (ICC: 0.75 (95% CI: 0.63, 0.84)) when observations with a House‐Brackmann score of I were excluded.

**Table 3:** Agreement between clinician‐administered and parent‐administered House‐Brackmann scores overall and at each follow up visit excluding cases where the clinician‐reported House‐Brackmann score was = 1

	Total sample			Aged < 12 y			Aged ≥ 12 y		
	N	ICC	(95% CI)	N	ICC	(95% CI)	N	ICC	(95% CI)
All time points^a^	172	0.73	(0.67, 0.78)	117	0.72	(0.65, 0.78)	55	0.75	(0.63, 0.84)
Baseline	172	0.53	(0.43, 0.64)	117	0.50	(0.36, 0.63)	55	0.60	(0.42, 0.75)
1 mo	79	0.81	(0.72, 0.87)	55	0.78	(0.66, 0.87)	24	0.86	(0.73, 0.94)
3 mo	19	0.46	(0.17, 0.78)	12	0.39	(0.08, 0.83)	7	0.27	(0.01, 0.92)

Abbreviations: CI, confidence interval; ICC, intraclass correlation coefficient.

^a^ ICC not estimatable at 6 months due to the small sample size.

**Figure 4:**
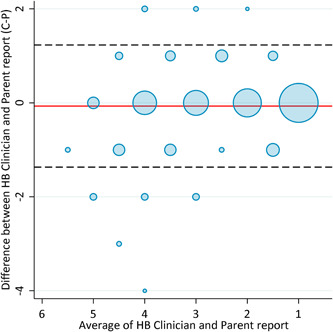
Bland‐Altman plot comparing clinician‐administered House‐Brackmann scores to the parent‐administered House‐Brackmann scores for the evaluation of facial nerve paralysis. The plot displays the difference between the values measured by the clinician and the parent‐administered House‐Brackmann scores against the mean of these values. The mean difference is represented by the horizontal red line and the 95% limits of agreement are shown by the dashed lines. The size of the circles indicates the frequency weights.

A Bland‐Altman plot (Figure 4) showed a mean difference between the 2 scores (clinician minus parent) of −0.07 with 95% limits of agreement of −1.37 to 1.23. The plot indicates excellent agreement between the 2 scales, with a potential negative bias at the higher values.

## Discussion

In this study, we investigated the agreement between assessment of facial deficits using a newly developed parent‐administered House‐Brackmann scale and the clinician‐administered House‐Brackmann scale. To our knowledge, this has not been done before. Overall, we found good agreement between parents and clinicians; however, agreement was poorer at baseline, improving at the follow‐up visits. These findings support the use of the parent‐administered House‐Brackmann scale as an estimation of the progress of a child's facial deficit during follow up if face‐to‐face follow up is not feasible. The agreement pattern was similar in children aged <12 years and ≥12 years.

The stronger agreement between the scales during follow‐up visits at 1, 3, and 6 months as compared to the agreement at baseline may be due to greater variability and more severe disease initially in the disease process. A study assessing clinician agreement of 2 facial grading scales, House‐Brackmann and Sunnybrook scales, also found lowest agreement at baseline compared to at follow‐up assessments.^18^ In both studies, the variability in the clinician‐reported scores at baseline may also have been due to this scale being administered in the ED by clinicians who varied in their seniority and experience in assessing FNP, whereas subsequent follow‐up visits were completed by a small number of specialist clinicians who were more experienced with the assessment of facial nerve disorders.^18^ Greater variability in the assessment of facial function has been reported between senior clinicians compared to juniors and medical students.^19^, ^20^ Increasing familiarity with the scales and greater familiarity with facial movement deficits by parents may also explain the improving agreement over time. In our study some of the follow‐up assessments may have been conducted by a different caregiver from the one who completed the baseline assessment.

In our post‐hoc analysis, we found that agreement dropped to moderate when children who were fully recovered (clinician rated House‐Brackmann grade of I) were removed from the analysis, which suggests that agreement improves in the absence of disease. In a study by Berg et al^21^ in adults with FNP who had House‐Brackmann score II to VI, the authors similarly reported moderate agreement between the House‐Brackmann and Sunnybrook scale, measured by weighted *K* levels of 0.54 and Spearman correlation coefficient of 0.76.

Overall, the moderate association between clinician‐ and parent‐reported measures of severity of FNP at follow up visits in children with active disease supports the use of parent report when a gross estimate of facial deficit is needed. For example, since most children fully recover before 3 months, the parent‐administered House‐Brackmann scale may be useful as a screening tool for poor progression toward recovery for families who lack access to specialty services because of cost, distance, availability, or other reasons such as COVID‐19 pandemic related restrictions.^4^ The strongest agreement was observed when children who were fully recovered were included in the analysis, which suggests that parent report may be also be useful and cost‐effective to track time to complete recovery in research. Nevertheless, in children experiencing worsening or persistent deficits or additional symptoms, in person clinician assessment will be required to provide a full characterization of facial deficits and rule out possibly sinister alternative diagnoses.^15^

## Limitations

The main limitation of the current study is, that while the clinician administered House‐Brackmann scale is used most frequently, there is no gold standard to assess the degree of FNP in children. The parent‐administered House‐Brackmann scale was not a validated tool and may have simplified the description of the facial deficits. We did not conduct repeat assessment by the same clinicians, by an additional independent observer or by a second parent to assess intra‐ or interobserver reliability. This shortcoming may be particularly relevant for the initial clinician assessment in the ED where junior clinicians may have graded participants.^19^, ^20^ We only assessed agreement between the scales in children with Bell's palsy though the results are likely applicable to other causes of FNP. The parent‐administered House‐Brackmann scale will need to be externally validated to support its broader use.

## Conclusions

Our data suggest that facial deficits rated by parents using a newly developed modified parent‐administered House‐Brackmann scale appear consistent with clinician assessment. Overall, there was good agreement between parents and clinicians in children with FNP, with correlation lowest at the initial assessment in ED and improving thereafter. While a thorough assessment of facial deficits requires clinician review, where such review is not possible, our data suggest that parent reports can provide clinically useful information on the progress towards recovery.

## Acknowledgments

We thank participating families and emergency department staff from participating sites. We thank research staff from the following sites: Andrew Davidson, John Cheek, Hannah Elborough, Donna Legge, Gill Ormond, Danica Van Den Dungen, Ashlea Logan, Cat Robson, Sarah Clark, Jenny May, Ashley Hill, Nicole Stromiloff (Murdoch Children's Research Institute, Melbourne, Victoria, Australia); Sharon O'Brien (Perth Children's Hospital, Perth, Western Australia, Australia); Gaby Nieva (Women and Children's Hospital, Adelaide, South Australia, Australia); Karthik Velusamy, Leonie Jones (The Townsville Hospital, Townsville, Queensland, Australia); Sally Gray, Gabrielle Taylor, Angus Jones, Mark O'Grady (Queensland Children's Hospital, Brisbane, Queensland, Australia); Adam West, Emma Ramage (Monash Medical Centre, Melbourne, Victoria, Australia); Shane George, Joanna Cronin (Gold Coast University Hospital, Southport, Queensland, Australia); Jason Hort, Deepali Thosar (The Children's Hospital at Westmead, Sydney, New South Wales, Australia); Ben Lawton, Vanessa Funk, Brooke Charters (Logan Hospital, Brisbane, Queensland, Australia), Frank Sullivan (University of St Andrews, School of Medicine, Edinburgh, United Kingdom) and Stuart Dalziel, Megan Bonisch, Jodie Livesy (Starship Hospital, Auckland, New Zealand).

## Author Contributions

**Franz E. Babl**, drafting/revision of the manuscript for content, including medical writing for content; major role in the acquisition of data; study concept or design; analysis or interpretation of data; **Nitaa Eapen**, drafting/revision of the manuscript for content, including medical writing for content; study concept or design; analysis or interpretation of data; **David Herd**, drafting/revision of the manuscript for content, including medical writing for content; major role in the acquisition of data; study concept or design; analysis or interpretation of data; **Meredith L. Borland**, drafting/revision of the manuscript for content, including medical writing for content; major role in the acquisition of data; study concept or design; analysis or interpretation of data; **Amit Kochar**, drafting/revision of the manuscript for content, including medical writing for content; major role in the acquisition of data; study concept or design; analysis or interpretation of data; **Michael Zhang**, drafting/revision of the manuscript for content, including medical writing for content; major role in the acquisition of data; study concept or design; analysis or interpretation of data; **Ed Oakley**, drafting/revision of the manuscript for content, including medical writing for content; major role in the acquisition of data; study concept or design; analysis or interpretation of data; **Sandy M. Hopper**, drafting/revision of the manuscript for content, including medical writing for content; study concept or design; analysis or interpretation of data; **Robert G. Berkowitz**, drafting/revision of the manuscript for content, including medical writing for content; study concept or design; analysis or interpretation of data; **Catherine L. Wilson**, drafting/revision of the manuscript for content, including medical writing for content; study concept or design; analysis or interpretation of data; **Amanda Williams**, drafting/revision of the manuscript for content, including medical writing for content; major role in the acquisition of data; study concept or design; analysis or interpretation of data; **Mark T. Mackay**, drafting/revision of the manuscript for content, including medical writing for content; major role in the acquisition of data; study concept or design; analysis or interpretation of data; **Katherine J. Lee**, drafting/revision of the manuscript for content, including medical writing for content; study concept or design; analysis or interpretation of data; **Stephen Hearps**, drafting/revision of the manuscript for content, including medical writing for content; study concept or design; analysis or interpretation of data.

## Disclosures

### Competing interests

None.

### Funding source

The study was funded by a grant from the National Health and Medical Research Council (NHMRC, project grant GNT1078069), Canberra, Australia, the Emergency Medicine Foundation (EMSS‐312R26‐2016‐GEORGE), Brisbane, Australia and the Perth Children's Hospital Foundation project grant #9670, Perth, Australia. The PREDICT research network was part funded by an NHMRC Center of Research Excellence grant (GNT1058560), Canberra, Australia, the Murdoch Children's Research Institute, Melbourne, Australia, and the Victorian Government's Operational Infrastructure Support program. FEB's time was part funded by a grant from the Royal Children's Hospital Foundation, Melbourne, Victoria, Australia and an NHMRC Practitioner Fellowship. Aspen Australia (St Leonards NSW 2065, Australia) provided the study drug (prednisolone and taste matched placebo) as a donation free of charge. Aspen did not sponsor the study and had no influence on study design, execution, analysis and publication.

## References

[1] Gilden DH . Bell's palsy. N Engl J Med. 2004;351(13):1323‐1331. 10.1056/NEJMcp041120 15385659

[2] Mackay MT , Chua ZK , Lee M , et al. Stroke and nonstroke brain attacks in children. Neurology. 2014;82(16):1434‐1440. 10.1212/wnl.0000000000000343 24658929

[3] Peitersen E . Bell's palsy: the spontaneous course of 2,500 peripheral facial nerve palsies of different etiologies. Acta Otolaryngol. 2002;122:4‐30. 12482166

[4] Babl FE , Herd D , Borland M , et al. Efficacy of prednisolone for bell palsy in children: a randomized, double‐blind, placebo‐controlled, multicenter trial. Neurology. 2022. 10.1212/wnl.0000000000201164 36008143

[5] Hotton M , Huggons E , Hamlet C , et al. The psychosocial impact of facial palsy: a systematic review. Br J Health Psychol. 2020;25(3):695‐727. 10.1111/bjhp.12440 32538540

[6] Saadi R , Shokri T , Schaefer E , Hollenbeak C , Lighthall JG . Depression rates after facial paralysis. Ann Plast Surg. 2019;83(2):190‐194. 10.1097/sap.0000000000001908 31232802

[7] Lee M , Mackay M , Blackbourn L , Babl FE . Emotional impact of Bell's Palsy in children: letters to the editor. J Paediatr Child Health. 2014;50(3):245‐246. 10.1111/jpc.12541 24674252

[8] Yılmaz Ü , Çubukçu D , Yılmaz TS , Akıncı G , Özcan M , Güzel O . Peripheral facial palsy in children. J Child Neurol. 2014;29(11):1473‐1478. 10.1177/0883073813503990 24097851

[9] House JW , Brackmann DE . Facial nerve grading system. Otolaryngol Head Neck Surg. 1985;93(2):146‐147. 10.1177/019459988509300202 3921901

[10] Sullivan FM , Swan IRC , Donnan PT , et al. Early treatment with prednisolone or acyclovir in Bell's palsy. N Engl J Med. 2007;357(16):1598‐1607. 10.1056/NEJMoa072006 17942873

[11] Federico A , Shi D , Bradshaw J . Agreement between parental report and clinician observation of infant developmental skills. Front Psychol. 2021;12:734341. 10.3389/fpsyg.2021.734341 34795613 PMC8593390

[12] Lifland BE , Mangione‐Smith R , Palermo TM , Rabbitts JA . Agreement between parent proxy report and child self‐report of pain intensity and health‐related quality of life after surgery. Acad Pediatr. 2018;18(4):376‐383. 10.1016/j.acap.2017.12.001 29229566 PMC5936667

[13] Libertus K , Landa RJ . The Early Motor Questionnaire (EMQ): a parental report measure of early motor development. Infant Behav Dev. 2013;36(4):833‐842. 10.1016/j.infbeh.2013.09.007 24140841 PMC3858411

[14] Babl FE , Gardiner KK , Kochar A , et al. Bell's palsy in children: current treatment patterns in Australia and New Zealand. A PREDICT study: Bell's palsy. J Paediatr Child Health. 2017;53(4):339‐342. 10.1111/jpc.13463 28177168

[15] Babl FE , Kochar A , Osborn M , et al. Risk of leukemia in children with peripheral facial palsy. Ann Emerg Med. 2021;77(2):174‐177. 10.1016/j.annemergmed.2020.06.029 32788067

[16] Babl FE , Mackay MT , Borland ML , et al. Bell's Palsy in Children (BellPIC): protocol for a multicentre, placebo‐controlled randomized trial. BMC Pediatr. 2017;17(1):53. 10.1186/s12887-016-0702-y 28193257 PMC5307816

[17] Lee K . *Bell's Palsy in Chlidren: Statistical Analysis Plan*. Murdoch Childrens Research Institute. Accessed November 18, 2022. https://mcri.figshare.com/articles/online_resource/Bell_s_Palsy_in_Chlidren_Statistical_Analysis_Plan/14708235

[18] Kanerva M , Jonsson L , Berg T , et al. Sunnybrook and House‐Brackmann systems in 5397 facial gradings. Otolaryngol Head Neck Surg. 2011;144(4):570‐574. 10.1177/0194599810397497 21493237

[19] van Veen MM , Bruins TE , Artan M , Werker PMN , Dijkstra PU . Learning curve using the Sunnybrook Facial Grading System in assessing facial palsy: an observational study in 100 patients. Clin Otolaryngol. 2020;45(5):823‐826. 10.1111/coa.13574 32419362 PMC7496591

[20] Reitzen SD , Babb JS , Lalwani AK . Significance and reliability of the House‐Brackmann grading system for regional facial nerve function. Otolaryngol Head Neck Surg. 2009;140(2):154‐158. 10.1016/j.otohns.2008.11.021 19201280

[21] Berg T , Jonsson L , Engstrom M . Agreement between the Sunnybrook, House-Brackmann, and Yanagihara facial nerve grading systems in Bell's palsy. Otol Neurotol. 2004;25(6):1020‐1026. 15547437 10.1097/00129492-200411000-00027

